# Parasitological cure and clinical benefits of benznidazole treatment in patients from the Jequitinhonha Valley, MG, Brazil, with recent chronic infection by *Trypanosoma cruzi* II

**DOI:** 10.1590/0074-02760240229

**Published:** 2025-06-02

**Authors:** Marta de Lana, Lourena Tomazelli Suave, Júlio César Santoro de Oliveira Assis, Girley Francisco Machado de Assis, Matheus Marques Milagre, Glaucia Diniz Alessio, Renato Afonso Salgado, Olindo Assis Martins-Filho, Pedro Albajar-Viñas, Rosália Morais Torres

**Affiliations:** 1 Universidade Federal de Ouro Preto, Núcleo de Pesquisas em Ciências Biológicas, Programa de Pós-Graduação em Ciências Biológicas, Ouro Preto, MG, Brasil; 2 Universidade Federal de Juiz de Fora, Laboratório de Parasitologia, Governador Valadares, MG, Brasil; 3 Universidade Federal de Ouro Preto, Programa de Pós-Graduação em Ciências Farmacêuticas, Ouro Preto, MG, Brasil; 4 Fundação Oswaldo Cruz-Fiocruz, Instituto de Pesquisas René Rachou, Grupo Integrado de Pesquisas em Biomarcadores, Belo Horizonte, MG, Brasil; 5 Hospital Nossa Senhora das Graças, Sete Lagoas, MG, Brasil; 6 World Health Organization, Department of Control of Neglected Tropical Diseases, Geneva, Switzerland; 7 Universidade Federal de Minas Gerais, Faculdade de Medicina, Belo Horizonte, MG, Brasil

**Keywords:** Chagas disease, benznidazole treatment, Jequitinhonha Valley, recent chronic infection, parasitological cure, clinical benefits

## Abstract

**BACKGROUND:**

The treatment of the early chronic phase of Chagas disease (CD) may result in high rates of parasitological cure, which may be associated with clinical benefits.

**OBJECTIVES:**

To evaluate children with CD from the Jequitinhonha Valley, MG, Brazil, treated with benznidazole (BZ), employing classic and alternative methodologies.

**METHODS:**

Before and after treatment, nine individuals were examined by haemoculture, polymerase chain reaction (PCR), conventional enzyme-linked immunosorbent assay (ELISA), electrocardiogram, echocardiogram, and thoracic and gastrointestinal X-ray. Eight individuals were in the indeterminate clinical form of CD, and one was in the mild cardiac form. After treatment, all individuals were re-evaluated periodically for 4-26 years using the same methodologies cited and anti-live trypomastigotes antibodies by flow-cytometry-FC-ALTA and quantitative PCR (qPCR).

**FINDINGS:**

The cure rate by the classic cure criteria was 33.33%. By the alternative cure criteria using FC-ALTA and qPCR, the rates of cure were 50% and 78%, respectively. Post-treatment clinical evaluations revealed stability in 5/9 and discrete clinical evolution in 4/9 individuals.

**MAIN CONCLUSIONS:**

It was demonstrated the effectiveness of BZ treatment in recent chronic infections of CD with low or higher rates of parasitological cure according to the cure criterion used after long-term follow-up. The clinical status of the individuals remained stable or evolved slowly, suggesting clinical benefits from BZ treatment.

Chagas disease (CD), caused by the etiological agent *Trypanosoma cruzi* (*T.cruzi*), is an important disease endemic in 21 Latin-American countries transmitted by triatomine vectors and several other mechanisms.^1^ It persists as a global and neglected public health problem with high morbidity and mortality burden. According to,^2^ ^,^ ^3^ 6-7 million people are infected worldwide, with approximately 12,000 disease-related deaths and 30,000-40,000 new cases per year. CD epidemiology has changed considerably in recent years as a consequence of the control measures adopted in the endemic countries and the frequent oral transmission of the disease by ingestion of contaminated foods.^4^ In addition, human migration of infected individuals to several countries on other continents has increased the transmission through mechanisms independent of the triatomine vectors.^5^

This disease may be asymptomatic in 60-70% of the infected individuals but develop severe cardiopathy in approximately 30% of the infected individuals, with the possibility of sudden death occurrence, as well as digestive alterations characterised mainly by megaoesophagus and/or megacolon, either associated or not with cardiopathy.^6^ ^,^ ^7^

Treating infected individuals with benznidazole (BZ) is a complementary measure that may offer some benefits to patients. However, this subject has been discussed in the BENEFITS project,^8^ ^,^ ^9^ which is still in progress. Furthermore, the treatment, even when not successful, reduces the parasitaemia and consequently several mechanisms of CD transmission other than the vectorial transmission, such as blood transfusion, vertical transmission, and organ transplantation.^10^

The possibilities of treatment for CD are limited to two drugs, nifurtimox and BZ.^11^ Both have great success in curing during the acute phase of the infection (70 to 100%), regardless of the transmission mechanism; relative success (approximately 60%) in recent chronic infections or patients aged 12 to 14 years,^12^ ^,^ ^13^ ^,^ ^14^ and less effectiveness in later chronic infections, where cure rates varied from 0 to 20%.^13^ ^,^ ^15^ Remarkably, the treatment of recent chronic infection has been recommended due to the rapid and greater decrease of parasitaemia, which indirectly affects *T. cruzi* transmission by vectorial and non-vectorial mechanisms, and mainly because it decreases the probability of clinical progression of the disease.^16^ ^,^ ^17^ ^,^ ^18^ However, studies differ in their casuistic related to age or time of infection, period of post-treatment evaluation, geographical origin, and the discrete typing unit (DTU) of *T. cruzi* involved in the infection,^19^ ^,^ ^20^ ^,^ ^21^ as well as clinical and laboratory methodologies employed in post-treatment evaluations, and interpretation of results, which together decisively influence the results obtained.^16^ ^,^ ^17^ ^,^ ^18^ ^,^ ^19^ ^,^ ^20^ ^,^ ^21^

Considering these points, in this study, we evaluated a group of children from two municipalities in the Jequitinhonha Valley, the second most important endemic region for CD in Brazil, who were diagnosed with *T. cruzi* infection and treated with BZ employing classic^22^ and alternative methodologies,^23^ ^,^ ^24^ including quantitative polymerase chain reaction (qPCR), through long-term evaluation after treatment.

## SUBJECTS AND METHODS

**Fig. 1:**
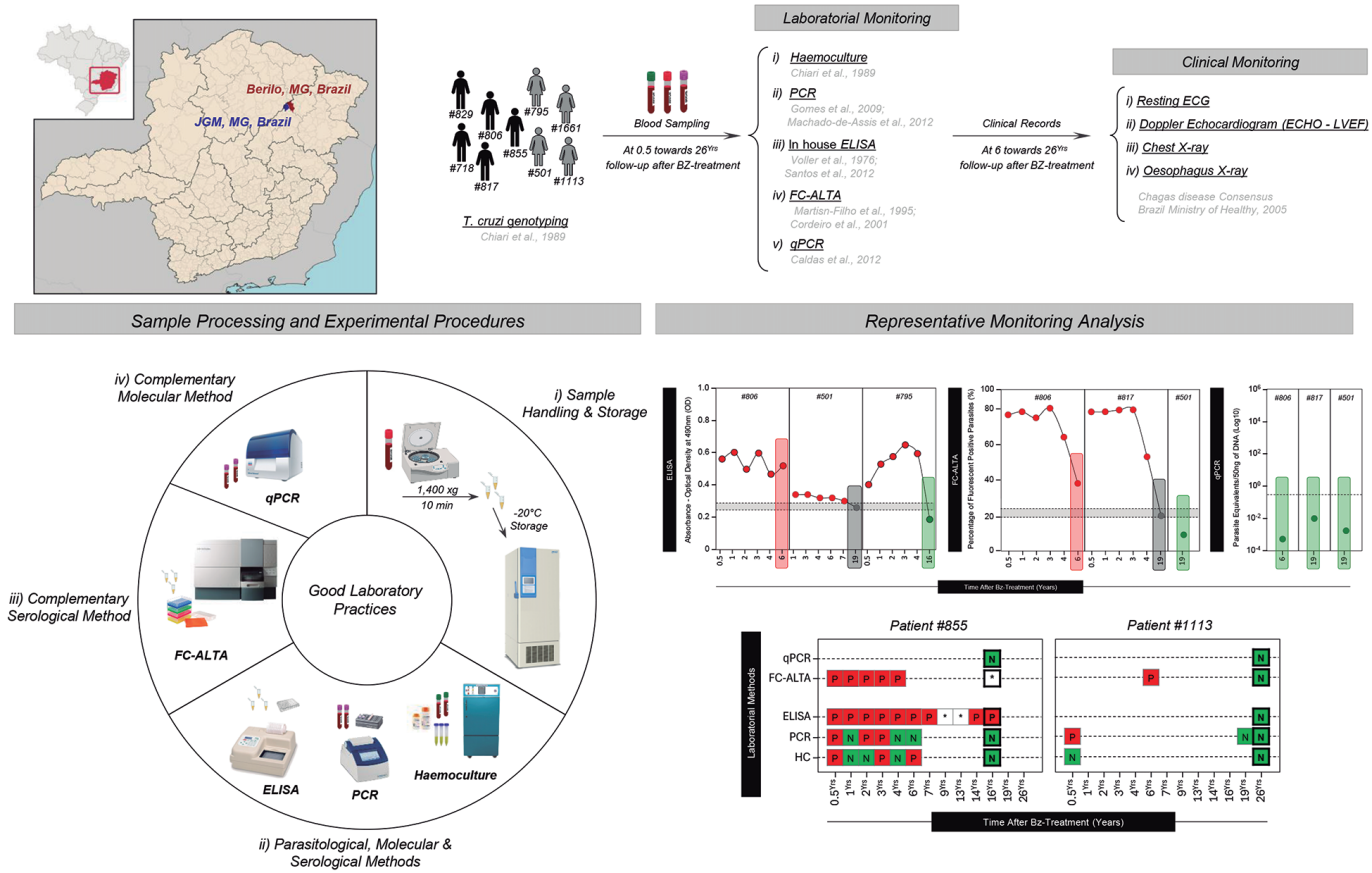
Chagas disease patients - Prospective laboratory and clinical follow-up after benznidazole (BZ) treatment.

*Patients description* - The total number of individuals in this study is nine (Table I). Six were diagnosed with *T. cruzi* infection during a serological inquiry for CD in 2002 in two municipalities, Berilo and José Gonçalves de Minas, MG State, located in the second most important endemic region of CD in the Jequitinhonha Valley region. All were treated in 2003 with BZ.^25^ In addition, three other children from Berilo were later included. One of these children had a case of vertical transmission of CD and was also treated with BZ. All individuals were serologically positive in in-house enzyme-linked immunosorbent assay (ELISA),^26^ and the reactivity was confirmed by IIF (Biomanguinhos) or HAI from Bio-Mérieux. Before treatment, parasites from 8/9 individuals were isolated and genetically characterised as TcII DTU^27^ ^,^ ^28^ (Table I). The individuals in this study consisted of five males and four females, six from Berilo and three from José Gonçalves de Minas municipalities, aged between seven and fifteen years old (Table I), when treated using the classical scheme for BZ (Roche^®^) therapy.^29^ A daily dose of 8 mg/kg of body weight was administered orally for 60 consecutive days.^25^ All individuals had the clinical form of the disease established before treatment: eight had the indeterminate clinical form of CD and one had the mild cardiac form. The study design (Fig. 1) shows the patients’ follow-up after treatment for four to 26 years.

**TABLE I:** Demographic, clinical features and discrete typing unit (DTU) genetic characterisation of Chagas disease patients treated with benznidazole (BZ) at recent chronic infection

Patient ID	Sex	Age at BZ-treatment (years)	Municipality	*Trypanosoma cruzi* DTU	Clinical form at BZ-treatment	Post-treatment follow-up (years)	Age at end of the follow-up
#829	Male	12	JGM	TcII	Indeterminate	4	16
#806	Male	11	JGM	TcII	Mild cardiac	6	17
#718	Male	7	Berilo	^*^	Indeterminate	9	16
#795	Female	13	Berilo	TcII	Indeterminate	16	29
#855	Male	13	JGM	TcII	Indeterminate	16	29
#1661	Female	15	Berilo	TcII	Indeterminate	16	31
#817	Male	9	Berilo	TcII	Indeterminate	19	28
#501	Female	15	Berilo	TcII	Indeterminate	19	34
#1113	Female	14	Berilo	TcII	Indeterminate	26	40

JGM: José Gonçalves de Minas; *not determined.

*Ethics* - All procedures were in accordance with the ethical standards of the responsible committee on human experimentation and with the Helsinki Declaration of 1975, as revised in 1983, and approved by the Ethical Committee at Instituto René Rachou (IRR), Belo Horizonte, MG, Brazil, Process 07/2002.

*Clinical evaluation before treatment* - Before treatment, all individuals were clinically evaluated by anamnesis and a clinical-cardiological examination. A resting electrocardiogram (ECG), chest X-ray in PA, radiologic study of the oesophagus, and Doppler echocardiogram were performed, and the clinical form of the disease was registered.

Patients were categorised as non-cardiac or chronic cardiac form. Patients with the non-cardiac form of CD had no clinical symptoms related to chronic Chagas heart disease, a normal electrocardiogram, chest X-ray, and no digestive symptoms. The clinical classification of Chagas heart disease was according to the Brazilian Consensus on Chagas Disease.^30^

A systematic and complete radiological evaluation of the entire gastrointestinal tract was not performed because the individuals were minors at the beginning of treatment and did not show any evidence of intestinal constipation or digestive symptoms that justified the use of barium enema.

*Treatment of the individuals* - Treatment was started only after the signing of informed written consent obtained from the individuals through their parents or legal guardians. The study was conducted with the utmost care and precision to ensure the safety and well-being of the individuals. Throughout the first month of treatment, the individuals were subjected to regular blood tests and biochemical analyses of hepatic and renal functions every 15 days to monitor their health condition. All patients completed the treatment without any significant side effects.

**Fig. 2:**
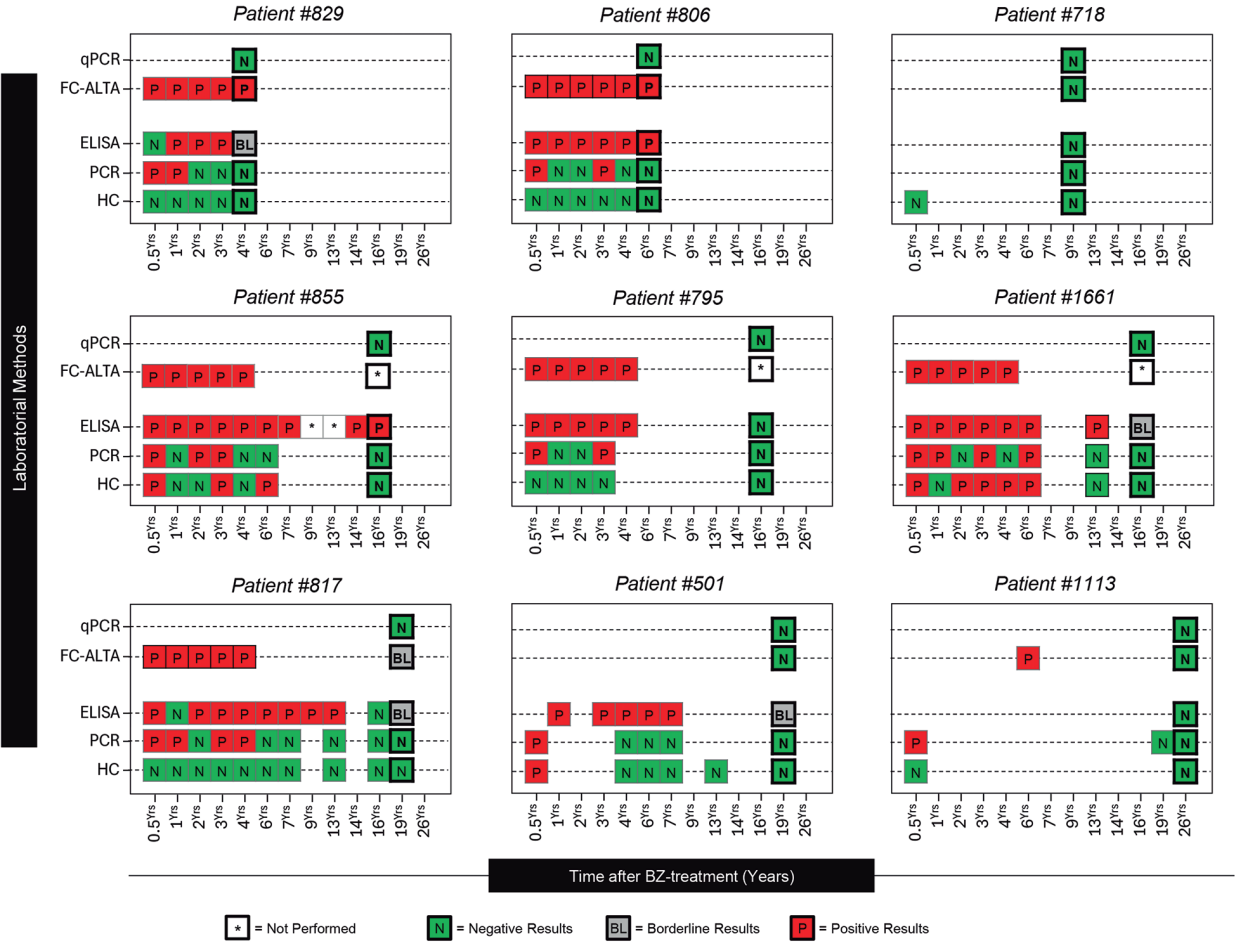
long-term follow-up of Chagas disease patients treated with benznidazole (BZ) in the recent chronic infection by parasitological (haemoculture), conventional serology enzyme-linked immunosorbent assay (ELISA), non-conventional serology (flow cytometry anti-live trypomastigote antibodies - FC-ALTA), and molecular polymerase chain reaction (PCR) and quantitative PCR (qPCR) methods.

*Post-treatment evaluations* - Several post-treatment evaluations (five to 11) were conducted from 2003 to 2023 (except in 2020 and 2021 due to the coronavirus disease 2019 - COVID-19 pandemic) in seven patients. Individuals 718 and 1113 were evaluated two to four times by at least one of the laboratory evaluations (Fig. 2).

*Parasitological tests* - Haemoculture (HC): for HC, a volume of 20 to 30 mL of blood, proportional to the body weight of all individuals, was processed.^31^

*PCR in blood eluate* - Five mL of venous blood was collected from each individual, and an equal volume of 6M Guanidine-HCl/0.2M EDTA was added (Sigma^®^ Chemical Company, USA). For DNA extraction, the “Wizard^®^ Genomic DNA Purification” and “DNeasy Blood & Tissue Kit (Qiagen Inc., USA) (Promega)” was used according to the manufacturer’s instructions. The reaction mixture used the primers Fw 121 (AAATAATGTACGGTGAGATGCATGA and Rv 122 GGTTCGATTGGGGTTGGTGTAATATA) (Invitrogen, São Paulo, Brazil), 6.25 μL of GoTaq^®^ Green Master Mix 2X (Promega, Madison, USA) and the amplification programme as described by^32^ and modified.^33^ As amplification control, all samples were amplified at the same plate with primers of the constitutive gene of human beta-globin (GAPDH), PCO3 (ACACAAACTGTGTTCACTAGC), and PCO4 (CAACTTCATCCACGTTCACC). The amplified products were visualised in electrophoresis using a 1% agarose gel stained with the DNA intercalator GelRed and a 100 bp molecular weight marker (QX SizeMarker^®^Qiagen), according to the manufacturer’s recommendations. Each sample was examined in duplicate in parallel with all reaction controls.

*qPCR reaction* - This test was performed only at each patient’s last evaluation (four to 26 years after treatment). DNA extraction of blood eluate used the same sample and protocol described for PCR. The qPCR technique was performed according to^34^ using the primers Fw TCZ-F 50-GCTCTTGCCCACAMGGGTGC-30 wherein M = A or C, and Rv TCZ-R 50-CCAAGCAGCGGATAGTTCAGG-30, which amplify a product of 182 bp, in the presence of SYBR Green PCR Mastermix “(Applied Biosystems). To quantify the parasite load by qPCR, a standard curve was constructed to determine the number of copies of the parasite DNA in the samples.^35^ The reaction was also performed to evaluate the GAPDH as an endogenous control employing the primers: forward 5’CTA CCC ACG GCA AAT TCC 30 and reverse 5’ACT CAG CAC CAG CAT CAC 30), and a 90 bp product was amplified (GENBANK: AB038240.1). The reactions were distributed in 96-well plates (Fast 96-Well Reaction Plate, 0.1 mL, MicroAmp TM). The amplified products were visualised in the QIAxcel Advanced System1 capillary electrophoresis (Qiagen), AM320 analysis method, 15 bp/600 bp alignment marker (QX Alignment Marker1, Qiagen), and 25 bp molecular weight marker 500 bp (QX Size Marker1 Qiagen), according to the manufacturer’s recommendations. The presence/absence of bands in the regions of interest of the electropherogram was obtained through QIAxcel ScreenGel 1.2.01 software (Qiagen). All samples were processed in duplicate in parallel to positive, negative and reaction controls.

Serological tests

*ELISA* - The ELISA technique used the methodology of^36^ modified by^26^ in sera diluted 1:80. For the reaction in microplates, crude antigen (4.5 µg/mL) from epimastigote of the Y *T. cruzi* strain, and peroxidase-labelled anti-human immunoglobulin G (Bethyl Laboratories, Montgomery, USA) were used. The enzyme-substrate (Ortho-phenylenediamine - OPD) was used for the reaction revelation. A reaction reading microplate (BIO-RAD, Model 3550) with a 490 nm filter was used. Ten negative and four positive control sera were added to each plate. The cut-off for each plate was the average absorbance of the ten negative sera plus two standard deviations. Samples with absorbance values below the cut-off were considered negative; when above, they were positive.

*Anti-live trypomastigotes antibodies by flow-cytometry (FC-ALTA)* - This test was performed in the first years after treatment in the six patients diagnosed in the serological inquiry and at nine (≠ 718), 19 (≠ 501), and 26 years (≠ 1113) after treatment. The methodology adapted for microplates and modified by using antigen of CL *T. cruzi* maintained in tissue culture was used.^23^ ^,^ ^24^ Briefly, sera samples were diluted in 96-well “U” bottom plates at 1:128, 1:256, and 1:512 and incubated with parasite suspension. For IgG1 analysis, the parasites were incubated again in 50 μL of biotinylated anti-human IgG1 antibody plus streptavidin conjugated with phycoerythrin-SAPE. Finally, parasites were fixed with 200 μL of fixative solution for cytometry (Max FacsFix-MFF paraformaldehyde, sodium cacodylate, and sodium chloride, pH 7.2). The reaction reading was on the flow cytometer [FACScalibur (BD Bioscience, San Diego, CA, USA)] using the CellQuest software version 3.3 for acquisition and data storage. For each test, internal control was used (conjugate control), where the parasites were incubated in water, without human serum, but in the presence of a secondary antibody to monitor non-specific binding. In all tests, positive and negative samples were included. The IgG1 reactivity was defined as the percentage of positive fluorescent parasites (PPFP). Samples were considered negative when PPFP < 20% and positive when PPFP > 20%.

*Cure criteria* - Three cure criteria were adopted: The classic cure criteria^22^ considered cured those patients with parasitological (HC and PCR) and serological examination (ELISA) negative post-treatment.

The alternative cure criteria^37^ considered cured patients with parasitological tests and non-conventional serological tests (FC-ALTA) negative even with the conventional serology still positive.

In addition, patients with qPCR, HC, and PCR negative in the presence of negative or residual reactivity of the conventional serology were considered parasitologically cured.

*Clinical evaluations* - In most post-treatment evaluations, the clinical examination was repeated whenever possible in parallel to laboratory evaluations to verify the impact of the BZ treatment on the clinical evolution of the disease.

*Electrocardiogram* - The ECG was performed safely, recording the twelve classic leads. Interpretation was carried out in accordance with the Safety Electrocardiogram Interpretation Guidelines.^38^

*Radiological assessment of the chest* - A radiological examination of the chest (chest X-ray) was performed in the posteroanterior (PA) view with the patient in a standing position and deep inspiration. The cardiothoracic index (CTI) was calculated by dividing the cardiac diameter (CD) by the thoracic diameter (TD), as measured on the posteroanterior chest radiograph. A CTI > 0.50 was considered abnormal and indicative of cardiomegaly, while a value < 0.50 was deemed normal. The hila, pulmonary vascular network, lung parenchyma, and pleura were assessed.^39^ ^,^ ^40^

*Echocardiogram* - A single specialist examiner conducted the Doppler echocardiogram using a Cypress portable echocardiography device. Two-dimensional and M-mode transthoracic echocardiography was performed at rest, complemented with pulsed and continuous colour Doppler, in line with the recommendations of the American Society of Echocardiography (ASE). The heart chambers were measured, and the left ventricular systolic and diastolic functions, as well as the left ventricle’s global and segmental contractility, were evaluated. Left ventricular systolic function was assessed by left ventricular ejection fraction using the Simpson’s method (left ventricular ejection fraction - LVEF) and M-mode. Throughout the systematic examination, we aimed to identify the presence of thrombi, aneurysms, valve regurgitation, pulmonary hypertension, and pericardial changes. The normality of left ventricular systolic function, as assessed by ejection fraction, was determined according to the ASE.^41^ The identification and staging of myocardial involvement adhered to the II Brazilian Consensus on Chagas Disease,^37^ based on data obtained from the ECG and the presence or absence of heart failure. Only the LVEF was considered in this study.

## RESULTS

*Parasitological evaluations after treatment* - The overall results of the parasitological evaluations are shown in Fig. 2. Six out of nine patients (# 829, # 718, # 806, # 795, # 817, # 1113) tested negative in HC repeated two to 10 times 0.5 to 26 years after treatment. Patient # 501 was positive only in the first evaluation 0.5 year after treatment, and # 855 and # 1661 had positive HC two to four times in the first six years after treatment, followed by negative results in the subsequent evaluations.

The PCR was positive in eight out of nine individuals assessed in the first evaluation post-treatment, followed by oscillating results (+/-) in four patients during the three to six years post-treatment (# 855, # 795, # 1661, and # 817), and consistently negative results in the subsequent evaluations for all patients examined after seven years post-treatment, as was observed in the HC (Fig. 2).

**Fig. 3:**
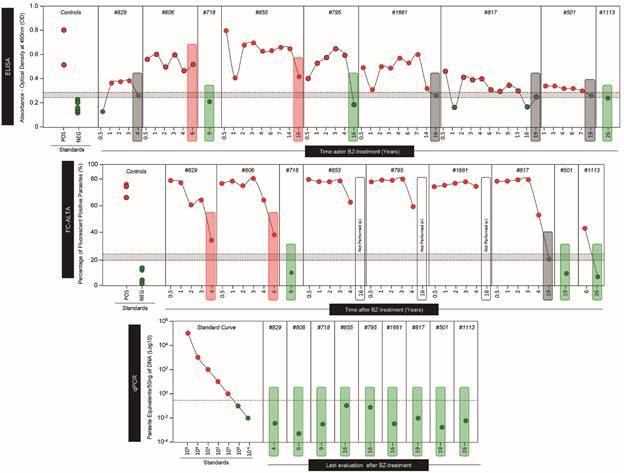
long-term follow-up of Chagas disease patients treated with benznidazole (BZ) in the recent chronic infection by conventional enzyme-linked immunosorbent assay (ELISA) and non-conventional (flow cytometry anti-live trypomastigote antibodies - FC-ALTA) serologies and molecular [quantitative polymerase chain reaction (qPCR)] methods.

The qPCR results conducted only in the final evaluation of all individuals were negative. In two individuals (# 795 and # 855), the results for k-DNA presence were residual (less than one parasitic cell) or due to dimers of the primers (Fig. 3, lower panel), which were consistent with or in agreement with the PCR negative in 100% of the individuals.

*Serological evaluations after treatment* - The ELISA test showed reactivity in several successive evaluations conducted in seven out of nine patients. The reactivity progressively declined during these evaluations, except for individual # 806 (Fig. 3, upper panel, and Fig. 2). Four patients (# 829, # 1661, # 817, and # 501) had borderline (BL) results in the final evaluations, and two (# 806 and # 855) remained reactive. ELISA turned negative in the last evaluation for three individuals (# 718, # 795, and # 1113), conducted nine, 16, and 26 years after treatment. Individual # 817 showed fluctuating results (-/BL) in the final two evaluations. In summary, 33.33% (3/9) of the treated patients tested negative in ELISA.

The non-conventional or alternative serology (FC-ALTA), conducted in the first four or five years post-treatment in six patients, was positive for all of them (Fig. 2, upper panel and Fig. 3, middle panel), with decreasing reactivity, particularly in patients # 829, # 806, and # 817. A subsequent evaluation of FC-ALTA at the 19th year of follow-up revealed that only patient # 817 exhibited BL reactivity. Patients # 718, # 501, and # 1113, who were examined only at nine, 19, and 26 years after treatment, respectively, tested negative using this technique.

*Final interpretation of treatment results* - Table II summarises the results of the latest laboratory evaluation (HC, PCR, ELISA, FC-HIGH and qPCR) used to monitor the parasitological cure of patients with CD following long-term follow-up.

**TABLE II:** Laboratorial records used for cure criteria monitoring of Chagas disease patients upon long-term follow-up after treatment with benznidazole (BZ) at recent chronic infection

Patient ID	Follow-up (years)	Laboratorial methods^*^					1st criteria (HC, PCR, ELISA)	2nd criteria (HC, PCR, ELISA & FC-ALTA)	3rd criteria (HC, PCR, ELISA & qPCR)
		HC	PCR	ELISA	FC-ALTA	qPCR				Age at end of the follow-up
#829	4	N	N	BL	P	N	16	ICP	Not cured		Cured
#806	6	N	N	P	P	N	17	Not cured	Not cured	Not cured
#718	9	N	N	N	N	N	16	Cured	Cured	Cured
#795	16	N	N	N	^**^	N	29	Cured	^**^	Cured
#855	16	N	N	P	^**^	N	29	Not cured	^**^	Not cured
#1661	16	N	N	BL	^**^	N	31	ICP	^**^	Cured
#817	19	N	N	BL	BL	N	28	ICP	ICP	Cured
#501	19	N	N	BL	N	N	34	ICP	Cured	Cured
#1113	26	N	N	N	N	N	40	Cured	Cured	Cured

BL: borderline results; ELISA: enzyme-linked immunosorbent assay; FC-ALTA: flow cytometry anti-live trypomastigote antibodies; ICP: in cure process; N: negative results; P: positive results; PCR: polymerase chain reaction; qPCR: real time PCR; ^*^ HC: haemoculture; ^**^ not performed.

According to the classic cure criteria,^22^ 33.33% of the patients were cured, which corresponds to individuals # 718, # 795, and # 1113. Four patients (# 829, # 1661, # 817, and # 501) were in the process of being cured (BL results) in the last evaluations and showed a decline in reactivity. Individuals # 806 and # 855 were not cured.

When the alternative cure criteria^37^ were considered, the rate of parasitological cure of the children treated and examined by CF-ALTA was 50% (3/6), corresponding to patients # 718, # 501, and # 1113. Individual # 817 was in the cure process (ICP), showing BL reactivity in ELISA and FC-ALTA. The other individuals were not examined later by FC-ALTA due to serum degradation.

In addition, according to the alternative cure criterion proposed here, patients with qPCR negative in the presence of negative conventional serology or decreasing reactivity are considered cured. Thus, 7/9 individuals are parasitologically cured, resulting in a cure rate of 78%.

*Clinical evaluations after treatment* - A percentage of 67% (6/9) of individuals were clinically stable after treatment (Table III). Three out of nine individuals progressed clinically from the indeterminate clinical form of the disease to cardiac. Individual 829 changed from the indeterminate to the digestive form of the disease, megaoesophagus, detected by contrast X-ray when evaluated six years after treatment. This patient did not return for new evaluations. The ECG changes observed in patients who progressed to the cardiac form were left anterior fascicular block, QRS deviation to the left, incomplete right branch lock, first-degree AV block, and extrasystoles. The chest X-ray showed a borderline cardiac index in two. The left ventricular ejection fraction (LVEF) was normal in all patients.

**TABLE III:** Clinical status of Chagas disease patients before and at long-term follow-up after benznidazole (BZ) treatment at recent chronic infection

Patient ID	Clinical form at BZ-treatment	Clinical follow-up (years)	ECG after BZ-treatment	ECHO (LVEF) after BZ-treatment	Chest X-ray after BZ-treatment	Clinical form at last evaluation
#829	Indeterminate	4	Normal	Normal	Borderline	Digestive (megaoesophagus^*^ )
#806	Mild cardiac	6	1º degree AV block; arrhythmia	Normal	Borderline	Mild cardiac
#718	Indeterminate	9	Normal	Normal	Normal	Indeterminate
#795	Indeterminate	16	Normal	Normal	Normal	Indeterminate
#855	Indeterminate	16	QRS complex left shift; left anterior fascicular block	Normal	Normal	Mild cardiac
#1661	Indeterminate	16	incomplete right bundle branch block	Normal	Normal	Mild cardiac
#817	Indeterminate	19	Normal	Normal	Normal	Indeterminate
#501	Indeterminate	19	Normal	Normal	Normal	Indeterminate
#1113	Indeterminate	26	Normal	Normal	Borderline	Indeterminate

AV: atrioventricular; ECG: electrocardiogram; ECHO: echodopplercardiogram; LVEF: left ventricular ejection fraction; ^*^ oesophagus X-ray.

## DISCUSSION

The purpose of this study was to verify the effect of BZ treatment in recent chronic infections on parasitaemia and disease evolution. This study evaluated a group of children infected with *T. cruzi* II DTU, which is very common in the central region of Brazil,^42^ particularly in the two municipalities of Berilo and José Gonçalves de Minas^24^ ^,^ ^25^ in the Jequitinhonha Valley, MG, Brazil.

The proper monitoring of patients with CD after etiological treatment is labour-intensive due to the need for serial evaluations over the years, and the use of several techniques,^43^ most of which are only employed in specialised CD laboratories, making them unfeasible for public health services. Parasitological tests with varying sensitivity and specificity, such as haemoculture,^31^ PCR,^44^ and qPCR,^45^ have been used in conjunction with conventional serological tests [ELISA, indirect immunofluorescence (IIF), and indirect haemagglutination (IHA)], which are the only ones available to public health services for monitoring parasitological cure. Additionally, non-conventional serological tests for detecting lytic antibodies^37^ or their analogues, such as the research of IgG anti-live trypomastigote antibodies (CF-ALTA),^23^ ^,^ ^24^ offer the advantage of allowing earlier determination of parasitological cure for *T. cruzi* infection when negative.

Thus, nine children treated for early chronic infection were monitored using the techniques previously mentioned. Six of them were evaluated consecutively at 0.5-19 years, and two patients were evaluated two to three times, at least in one, two, or all the evaluations, at 0.5 to 26 years after treatment.

The haemoculture was negative, as expected due to its low sensitivity,^31^ ^,^ ^46^ including in patients who had been etiologically treated from the same municipalities^33^ ^,^ ^47^ ^,^ ^48^ and examined long after treatment. From the first examination, six months after treatment, 7/9 patients tested negative and remained negative in all subsequent evaluations from the seventh year following treatment. During this same period, PCR was positive in all patients, with an increasing number of positive results for each one. Even with higher sensitivity,^44^ PCR showed the same final outcome as haemoculture, with negative results from the seventh year following treatment, suggesting a drastic reduction in parasitaemia in all patients. Interestingly, qPCR, with higher sensitivity and specificity under our experimental conditions, and the most potent technique indicative of treatment failure when positive,^45^ performed in the final evaluation of all individuals, was negative in all patients, showing residual reactivity of < 0.1 k-DNA, equivalent to one parasite, in two individuals. Regarding the results of conventional serology (ELISA), which slowly decreases in reactivity,^49^ particularly in patients from the studied region,^33^ ^,^ ^47^ ^,^ ^48^ only 3/9 individuals were negative (three patients) or had borderline reactivity (four patients) in the final evaluation. This result demonstrated the gradual reduction in reactivity of this serology, as described in the literature, particularly in patients treated etiologically during the chronic phase. However, in two of the individuals who were successively evaluated after treatment, ELISA was still positive, while four patients showed decreasing reactivity alongside negative parasitological tests (HC, PCR, and qPCR), indicating that these individuals were in the process of cure and require further evaluations.^50^

Although the alternative serology FC-ALTA was not conducted successively across all patients throughout the follow-up, a significant reduction in its reactivity was observed between the two evaluations (four or five years vs. 16 or 19 years of treatment), reaching 50% negative results in the final evaluation.

Unfortunately, comparing the results of conventional and non-conventional serology was not possible because patients who were evaluated at the beginning of the study either moved away from the region or failed to attend the final evaluation.

The overall results, with a group of patients predominantly infected with TcII, demonstrated treatment success long time after treatment, achieving a cure rate of 33.33% (3/9) by the classic cure criteria,^22^ which show negative results in HC, PCR, and ELISA. Three out of six individuals evaluated using FC-ALTA were considered cured according to the alternative cure criteria,^37^ ^,^ ^23^ ^,^ ^24^ with negative results in HC, PCR, and ELISA. According to this second cure criterion applied to six patients examined, 50% were cured. However, 78% of parasitological cures were detected by the alternative cure criteria using qPCR (individuals with negative qPCR, HC, PCR, and negative or residual reactivity in ELISA), reinforcing the idea that qPCR is the best biomarker for treatment failure when positive and suggest of cure when negative. This can be verified here because qPCR was used alongside other parasitological tests (HC and PCR)^44^ and conventional serology that presents late seroconversion. An interesting aspect is the compatibility between the three cure criteria used here, with the third one, using qPCR, identifying only one more patient as cured compared to the combination of the classic and first alternative cure criteria, which employed FC-ALTA. There was no association between the age at treatment or the follow-up time and the occurrence of parasitological cure, according to the three cure criteria used. The absence of these associations was likely due to the fact that, during the study, it was impossible to monitor all patients with all the laboratory tests.

The impact of BZ treatment on clinical evolution, as observed in several studies by different authors treating patients with early chronic infection,^16^ ^,^ ^17^ ^,^ ^19^ ^,^ ^51^ ^,^ ^52^ resulted in therapeutic success linked to clinical benefits following post-treatment follow-up. The hypothesis is that if Chagas’ cardiomyopathy is triggered by persistent parasitic infection, it is plausible that trypanocidal therapy could delay, reduce, or prevent disease progression.^12^ Consequently, children under 16 years old should be treated. In fact, our results showed similar findings in individuals treated between nine and 15 years old. Five out of nine remained in the indeterminate clinical form, while three clinically evolved to mild cardiopathy after four to 26 years of follow-up, analysed periodically using ECG and echocardiogram (LVEF). Interestingly, all patients who were cured based on the classic cure criteria^22^ and the alternative or second cure criteria^37^ ^,^ ^23^ ^,^ ^24^ remained in the indeterminate clinical form of CD. These results are consistent with immunological evaluations of six/nine patients before and one year after treatment, showing that BZ treatment led to substantial T and B-cell activation, associated with high IL-10 levels, which directed their activation state towards a modulated cytokine profile.^53^ These changes may explain the benefits of the etiological treatment of CD described here. The high cure rate, considering the two alternative cure criteria alongside clinical stability or low clinical progression in patients aged between 16 and 40 years (mean age 27 years at the last clinical evaluation), strongly suggests that these results, combined with a substantial reduction in parasitaemia, may lead to an immunological shift in the host immune response, which could keep the parasite under control until its complete elimination.

The later detection of parasitological cure several years after treatment with BZ, mainly by the classic cure criteria, particularly in individuals predominantly infected with *T. cruzi* II, is expected. Resistance to BZ and nifurtimox (NF) treatment has been observed in studies of patients from Central Brazil,^51^ ^,^ ^54^ where TcII is predominant.^42^ This reality contrasts with what has been observed in other countries in Central or South America, where TcI and TcIII are predominant and post-treatment seroconversion occurs early,^14^ ^,^ ^55^ ^,^ ^56^ or even in Chile and Argentina,^17^ ^,^ ^57^ where TcV and TcVI are predominant.^42^

Despite the results obtained, some limitations of this study must be considered, such as the absence of a control group of untreated individuals infected with *T. cruzi*, which would have been unethical, and the loss of follow-up for individuals who moved away from the region studied, and some degradation of sample sera. Even with these limitations in mind, we hope these results will encourage the administration of BZ to treat all children diagnosed with CD. Such practices will greatly contribute to the prevention of other mechanisms of CD transmission, along with the clinical benefits demonstrated, such as clinical stability or slow clinical evolution over 0.5-26 years of follow-up.

In conclusion, the results demonstrated the effectiveness of BZ treatment in recent chronic infections of CD, with both low and higher rates of parasitological cure, depending on the cure criterion adopted after long-term follow-up. The clinical status of the individuals remained stable or evolved slowly, suggesting clinical benefits from BZ treatment.
